# An end is in sight: a perspective on PCR as an endpoint for Chagas disease treatment trials

**DOI:** 10.3389/fpara.2023.1272386

**Published:** 2023-10-09

**Authors:** Natasha S. Hochberg, Srinivasa P. S. Rao, Gerhild Angyalosi, Xiaojun Zhao, Leticia Carballo, Caroline Demacq, Sofia Braud-Perez, Daniela Wieser, JP Casas, John Millholland, Debby Ngo

**Affiliations:** ^1^ Novartis Biomedical Research, Cambridge, MA, United States; ^2^ Novartis Biomedical Research, Emeryville, CA, United States; ^3^ Novartis Pharma AG, Basel, Switzerland

**Keywords:** *Trypanosoma cruzi* (*T. cruzi*), diagnostic test, efficacy, novel chemical entities, parasite

## Abstract

Novel therapies for chronic indeterminate Chagas disease (CICD) are needed, but trials are limited by the absence of tests to detect infection and early treatment efficacy. This perspective highlights the shortfalls and strengths of polymerase chain reaction (PCR) as a study endpoint for anti-parasitic drug development. Serologic reversion, the gold standard test of cure, may take decades to occur in adults and therefore is challenging as an endpoint for drug development. Use of PCR as a marker of infection and treatment response has notable limitations due to low parasitemia in CICD, fluctuations in circulating (versus tissue) parasite burden, strain differences, and assay performance. It is, however, rapidly responsive to therapy, and technological advances have improved detection of different strains and may allow for parasite quantification. Until we have more sensitive tests for parasitological clearance, PCR as a measure of treatment failure may be the best available efficacy endpoint to accelerate early development of much-needed novel therapies. Adequately designed clinical studies are needed to correlate PCR clearance with clinical outcomes and to identify novel biomarkers predictive of clinical outcomes in patients with CICD. Public-private partnerships and health authority engagement are paramount to identify feasible trial endpoints and deliver promising new drug candidates for Chagas disease.

More than 5 million individuals live with Chagas disease worldwide and a subset with chronic indeterminate Chagas disease (CICD) will develop potentially fatal complications (Hochberg and Montgomery, 2023). The standard of care therapies, benznidazole and nifurtimox, although effective at clearing the parasite, currently require 60 days of treatment and have frequent adverse reactions often requiring treatment discontinuation (Forsyth et al., 2016; Crespillo-Andujar et al., 2018). As novel therapies are being developed to treat Chagas disease (Khare et al., 2016; Saldivia et al., 2020; Padilla et al., 2022; Rao et al., 2023), clarity is needed on the registration endpoints acceptable to health authorities. This viewpoint provides a drug development perspective on the role of real-time polymerase chain reaction (PCR) as a measure of parasitemia and its utility as an outcome measure for treatment trials, in particular for CICD.

To date, PCR has been used as the primary efficacy measure in early trials of new regimens for benznidazole and nifurtimox as well as novel chemical entities (NCE) for CICD (Solari et al., 2001; Molina et al., 2014a; Molina et al., 2014b; Morillo et al., 2015; Morillo et al., 2017; Torrico et al., 2018; Torrico et al., 2021). It is a powerful tool for detecting low levels of circulating *Trypanosoma cruzi* DNA in patient blood samples. For testing the efficacy of direct anti-parasitic NCEs, blood PCR testing may build confidence that the compound is efficacious in reducing parasitemia. However, PCR also has several biological limitations. Circulating parasitemia may not reflect parasite levels in tissues; in fact, among individuals with CICD, only ~50-70% have a positive blood PCR test result before treatment (Molina et al., 2014a; Ramirez et al., 2015; Alvarez et al., 2016). Although a positive PCR indicates treatment failure, PCR clearance in blood does not necessarily equate to clearance of parasite from the tissues. Confidence in the test result may be increased with repeated PCR testing, as parasitemia may be intermittent in some CICD patients (Parrado et al., 2019). From a long-term perspective, although cardiomyopathy is associated with parasite persistence in cardiac tissue, it is not clear whether blood parasite clearance, measured by PCR, results in clinical benefit (e.g., decreased progression to cardiomyopathy) (Tarleton, 2003; Kierszenbaum, 2007; Bonney and Engman, 2008).

Despite advances in PCR technologies, including quantitative PCR, technical limitations remain. Assay sensitivity is affected by blood volume collected for DNA isolation, the extraction method, and the detection system; furthermore, there is an inability to differentiate DNA fragments after parasite death. The heterogeneity of the parasite genome, including the parasite discrete typing units (DTU), presents substantial challenges since primers and probes may not be able to detect all *T. cruzi* DTUs equally (Hagstrom et al., 2019; Torrico et al., 2021). From a clinical trial perspective, comparison of results has been hampered by variability in the timepoints of PCR assessment (e.g., sustained through 6 months versus 12 months), numbers of DNA extractions, and the different assays used (Parrado et al., 2019). A recent systematic review documented high variability across methods, further highlighting the challenges to compare results between studies (Hagstrom et al., 2019). Encouragingly, there has been increased attention paid to developing a standard metric recommended for use in studies to improve comparability (Schijman et al., 2011; Duffy et al., 2013; Ramirez et al., 2015; Munoz-Calderon et al., 2022). Commercial PCR diagnostic kits have been developed by several companies which may facilitate implementation across clinical laboratories. Continued efforts are needed to further standardize and validate methods in different populations to establish clear guidelines for interpretation and clinical application. Ultimately to validate PCR, engagement with health authorities would be needed; this step would be a significant advancement in clinical trials of Chagas disease.

Although the PCR assay has limitations, certain factors in its favor are worth noting. First, parasitemia is often higher among infected newborns (in peripheral or cord blood), immunosuppressed individuals, and those with acute Chagas disease, suggesting that in these scenarios, treatment failure may be confirmed by PCR. Similarly, PCR may be more relevant for those individuals with CICD who have a positive PCR test result before treatment (Murcia et al., 2010). Secondly, the use of PCR is used standardly for other infectious diseases. Viral load is measured for diseases such as hepatitis C and cytomegalovirus (Lawitz et al., 2013; Kimberlin et al., 2015). Even among other viruses for which variable and undetectable viral reservoirs may exist, like human immunodeficiency virus (HIV), viral load measurement is used (Group et al., 2019). Furthermore, after the rise of SARS-CoV-2 (COVID-19), improved and simplified technologies were developed to carry out PCR assays, and these have entered wide-scale use (Khamsi, 2022). Advances in digital droplet PCR (Pomari et al., 2019; Ramirez et al., 2019), isothermal amplification (Schijman, 2018; Munoz-Calderon et al., 2022), and next-gen sequencing (Kamath et al., 2020) hold additional promise for application in neglected tropical diseases. For Chagas disease in particular, newer PCR assays are overcoming limitations of older assays (Ramirez et al., 2015).

Steps can be taken to improve PCR as an endpoint for clinical trials and to identify other biomarkers that may meet an acceptable target product profile for CICD (Porras et al., 2015). First, well-curated, standardized longitudinal cohorts and biobanks, such as the SamiTrop and REDs-II cohorts are needed to enable correlation of short-term PCR reversion with longer-term serologic test results and clinical outcomes (Oliveira et al., 2021). Second, clinical trials should attempt to harmonize assays and endpoints. Third, resources are needed to develop PCR assays with improved sensitivity to detect low levels of circulating parasites across all strains. Additional efforts are also needed to identify alternate biomarkers that detect tissue parasites and correlate with long-term treatment outcomes. Novel serologic assays (e.g., MultiCruzi) are under investigation that may add value (Granjon et al., 2016; Jurado Medina et al., 2021).

In conclusion, PCR has to date played a critical role in proof-of-concept clinical studies using anti-parasitic agents. Not only is a positive PCR result an inclusion criterion in many clinical trials (Solari et al., 2001; Molina et al., 2014a; Molina et al., 2014b; Morillo et al., 2015; Morillo et al., 2017; Torrico et al., 2018), it can efficiently identify treatment failure with suboptimal drugs and is a valuable tool in the selection of the most promising candidates to advance to the next stage of drug development. With its rapid turn-around time and acceptance as a marker of treatment failure, PCR will likely continue to play a role in early phase studies to test anti-parasitic effects despite the fact that the correlation of PCR and long-term clinical outcomes is not clearly established. Alongside clinical drug development of NCEs, a path is needed to identify, validate, and approve a novel, feasible surrogate endpoint. A collective effort from the Chagas disease community, including academia, government, non-governmental agencies, and pharmaceutical industry partners, is urgently needed to address biomarker gaps. Looking forward to late phase and registrational trials, health authority feedback into acceptable endpoints for treatment efficacy will be crucial to lay the foundation for better therapies to treat Chagas disease.

## Data availability statement

The original contributions presented in the study are included in the article. Further inquiries can be directed to the corresponding author.

## Author contributions

NH: Conceptualization, Project administration, Writing – original draft, Writing – review & editing, Data curation. SR: Conceptualization, Data curation, Project administration, Supervision, Writing – review & editing. GA: Data curation, Writing – review & editing. XZ: Data curation, Writing – review & editing. LC: Data curation, Writing – review & editing. CD: Data curation, Writing – review & editing. SB-P: Writing – review & editing. DW: Data curation, Writing – review & editing. JC: Writing – review & editing. JM: Data curation, Writing – review & editing. DN: Conceptualization, Data curation, Writing – original draft, Writing – review & editing.
